# Community-applied research of a traditional Chinese medicine rehabilitation scheme on Broca’s aphasia after stroke: study protocol for a randomized controlled trial

**DOI:** 10.1186/1745-6215-15-290

**Published:** 2014-07-21

**Authors:** Jing Tao, Yunhua Fang, Zhenkai Wu, Ting Rao, Yusheng Su, Lili Lin, Wei Liu, Jinsong Wu, Shanli Yang, Guohua Zheng, Lidian Chen

**Affiliations:** 1 Rehabilitation Medicine College, Fujian University of Traditional Chinese Medicine, No.1 Huatuo Road Shangjie Minhou, Fuzhou 350122, China; 2 Academy of Integrative Medicine, Fujian University of Traditional Chinese Medicine, No.1 Huatuo Road Shangjie Minhou, Fuzhou 350122, China; 3 Fujian University of Traditional Chinese Medicine, No.1 Huatuo Road Shangjie Minhou, Fuzhou 350122, China

**Keywords:** Aphasia, Stroke, Traditional Chinese medicine, Rehabilitation, Randomized controlled trial

## Abstract

**Background:**

Aphasia is a common and severely disabling complication in stroke patients. It usually brings about lower rates of functional recovery, longer rehabilitation length of stay (LOS), and significantly poorer LOS efficiency (LOS-Eff), resulting in higher rehabilitation costs compared to patients without aphasia. It also decreases the quality of life and increases the mortality of stroke patients. The evidence currently available suggests that the effect of acupuncture combined with language training for apoplectic aphasia is statistically better than speech and language therapy (SLT) alone, but there remains a lack of high-quality randomized controlled trials. Acupuncture combined with language training is relatively low-cost and especially suitable for community-based rehabilitation for aphasia patients after stroke, taking its medical and health facilities which are always deficient in manpower and material resources into account. The aim of the present study is to develop an effective standard therapeutic program for apoplectic aphasia in communities.

**Methods/Design:**

In a randomized controlled clinical trial with blinded assessment, 290 eligible patients with aphasia due to stroke will be randomly allocated into a control group or an experimental group. The course of this trial will comprise a 4-week intervention and a 12-week follow-up period. Five assessment points, including baseline, 2 and 4 weeks after treatment, 6 and 12 weeks after follow-up, are set to dynamically observe the changes of curative effects. Primary outcome measures are the differences in the score on both the China rehabilitation research center aphasia examination (CRRCAE) and Boston diagnostic aphasia examination - Chinese version (BDAE-C) after intervention and follow-up. The Modified Barthel Index (MBI), 36-Item Short Form Health Survey (SF-36), and results of blood oxygen level dependent-functional magnetic resonance imaging (BOLD-fMRI) examination are considered as the secondary outcome measures. Other outcomes will include rate of adverse events and economic effects.

**Discussion:**

If the outcome is positive, this project will offer a low-cost appropriate technology for community health centers (CHCs) in the rehabilitation of aphasia patients after stroke, and could be implemented on a large scale, both in China and worldwide.

**Trial registration:**

Chinese Clinical Trial Registry: ChiCTR-TRC-13003703. Registration date: 18 October 2013.

## Background

Aphasia is a common complication in stroke patients, with lower rates of functional recovery [1,2], longer rehabilitation length of stay (LOS), and significantly poorer LOS efficiency (LOS-Eff) compared to patients without aphasia [3]. Furthermore, it proves that the occurrence of aphasia has an influence on the direct costs of rehabilitation [4]. Language dysfunction in the post-acute or chronic phase after a stroke is the major restriction of vocational rehabilitation [5] and reduces the probability of a return to work [6]. Aphasia is associated with emotional burden [7] and limits social participation owing to impaired communication ability [8], as well as with a decrease in the quality of life [9] and an increase in the mortality of stroke patients [10]. Thus, there is an urgent need to search for one method which is both effective and low-cost for the rehabilitation of stroke patients, especially those discharged from hospital back into the community.

Although most patients with post-stroke aphasia present some degree of spontaneous recovery, this tends to plateau by one year after onset [11]. Approximately 40 to 60% of patients move from the acute stage to the chronic stage if the condition persists 6 to 12 months after stroke [12]. Speech and language therapy (SLT), the effectiveness of which is documented in a Cochrane systematic review on SLT for people with aphasia following stroke [13], is the most common treatment [14]. SLT in the acute stages of apoplectic aphasia is almost twice as effective as natural recovery alone [15]. Unfortunately, current SLT has only limited effectiveness in improving aphasia, and other approaches adjunct to SLT might be used [16].

As a typical traditional Chinese medicine, acupuncture has been applied for thousands of years [17], and it has been widely used for aphasia rehabilitation in China. Many clinical studies about acupuncture therapy for aphasia have been conducted in the past few decades. It has been confirmed that acupuncture is an effective adjuvant therapy to SLT in the rehabilitation of aphasia [14]. To obtain a more credible evaluation of the effect of acupuncture for post-stroke aphasia, Pang *et al*. [18] performed a systematic review of the relevant randomized controlled trials, and the results support the conclusion that the effect of acupuncture combined with language training for apoplectic aphasia is statistically better than SLT alone. However, the included articles are assigned as low-quality by the authors, with a suggestion that more high-quality randomized controlled trials needed to be performed.

At present, professional rehabilitation of aphasia is offered mainly in high-grade hospitals, which are rich in interventional technologies but are relatively highcost. The heavy burden on families may be one of the major reasons for the early discharge of stroke survivors from hospitals [19]. A study by Klebic *et al*. [20] observed that only 21.2% of patients with aphasia continued language therapy after leaving hospital, which negatively affects functional recovery [21]. The provision of stroke rehabilitation services in the community is supported by evidence [19], and community-based rehabilitation for aphasia patients after stroke is especially important and urgently needed. Taking the medical and health facilities of community into account, the therapy we select for aphasia patients after stroke should be more effective, lowcost, convenient, and uncomplicated. Scalp acupuncture treatment, which meets the above requirements, will be a suitable selection.

The goal of the present study, combined with a randomized, parallel-controlled design, is to develop an efficacious and economical therapeutic program for apoplectic aphasia in communities. Finally, it will be implemented on a large scale if it is found to be an appropriate technology.

## Methods/Design

### Study design

**Figure 1:**
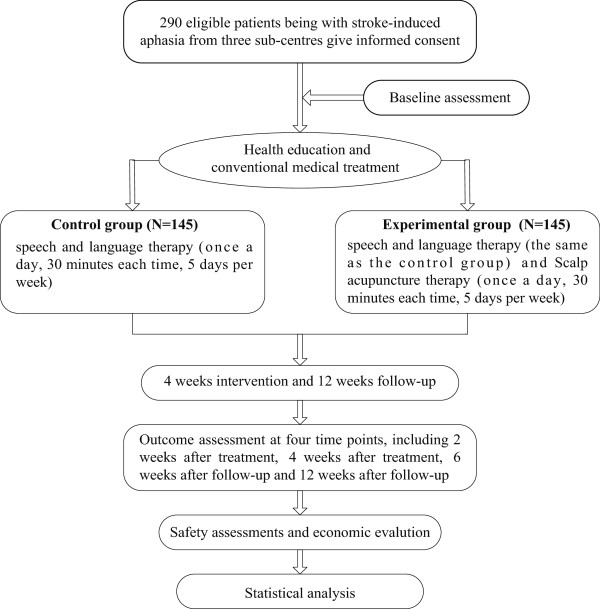
Flow diagram of study design.

The current study uses a multicentre, cluster randomized, parallel-controlled superiority design with blinded assessment (a flow diagram of the study design is presented in Figure 1). The course of this trial will comprise a 4-week intervention and a 12-week follow-up period. The control group will receive SLT and initial therapies, while, the experimental group will receive additional scalp acupuncture. There is a total of five time points, including baseline, 2 weeks after treatment, 4 weeks after treatment, 6 weeks after follow-up, and 12 weeks after follow-up. Fujian University of Traditional Chinese Medicine (FJTCM), as an undertaker of this project, is responsible for completing the training of the rehabilitation therapists in a unified standard way before the study, and supervising their operations regularly during the study at all clinical sites. The Center of Evidence-based Medicine in FJTCM, which plays a role in randomization and will be blinded to the intervention, is an independent department employed to monitor and analyze data. The present project, funded by the State Administration of Traditional Chinese Medicine, has received approval from the local ethics committees of every center (Fujian: 2013KY-006-01, approval received in July 2013; Henan: 2014HL010, approval received in March 2014; Shandong: 2013KY-006-01, approval received in March 2014) participated in the study. Written informed consent is required prior to participation from each participant or their guardian, and they will be informed of the nature of this trial including its purpose, procedures involved, expected duration, potential risks and discomfort, as well as the possible benefits they will receive from it. There is no time limit for them to ask related questions and respond to the invitation to participate. Participants will be also informed that they are free to withdraw from the study at any stage for any reason and their personal information will be undisclosed and kept securely at FJTCM.

### Participants and recruitment

The protocol will be carried out at community health centers (CHCs) run by three sub-centers, including the Affiliated Rehabilitation Hospital of Fujian University of Traditional Chinese Medicine (Fujian province, China), the Second Affiliated Hospital of Shandong University of Traditional Chinese Medicine (Shandong province, China), and the First Affiliated Hospital of Henan College of Traditional Chinese Medicine (Henan province, China). Many CHCs in different areas of the three provinces will be included in this protocol, providing a large number of patients.

### Inclusion criteria

The eligible participants should meet all the following criteria: (1) have been diagnosed with stroke according to the criteria regulated by the Fourth National Cerebral Vascular Disease Conference [22], and confirmed as first stroke by a computed tomography (CT) or magnetic resonance imaging (MRI)examination; (2) be no more than two years post-stroke; (3) suffer from Broca’s aphasia after stroke, determined by the neurologist or rehabilitation physician and speech and language therapist, ascertained with Chinese Rehabilitation Research Center Standard Aphasia Examination (CRRCAE); (4) be between 18 and 80 years of age; (5) have provided written informed consent.

### Exclusion criteria

Patients having anyone of the following will be excluded: (1) aphasia due to another encephalopathy, such as traumatic brain injury, brain tumor, neurodegenerative diseases, brain parasitic diseases and so on; (2) aphasia due to dementia or dysarthria; (3) stroke with serious uncontrolled complications, such as severe pulmonary infection, shoulder-hand syndrome or deep vein thrombosis; (4) having severe uncorrected to normal visual or auditory impairment; (5) having serious heart, kidney, liver, or nervous system disease; (6) having a history of epilepsy; (7) having received an alternate intensive intervention which may affect the evaluation results during the last 4 weeks;

### Sample size

Referring to a trial [23] with the same protocol as the present study, the effective rate of Broca’s aphasia after stroke was 64.71% in the control group (π_1_) and 85% in the experimental group (π_2_). Preliminary epidemiological findings show that there will not be less than 16 qualified participants recruited from each CHC. Prior data indicates that the intra-CHC correlation coefficient (ρ) is likely to be 0.030. Each group will consist of 132 individuals according to the formula:

(1) $$n=\frac{{\left(\mu_{\alpha }+\mu_{\beta }\right)}^{2}\;\left[\pi_{1}\;\left(1-\pi_{1}\right)+\pi_{2}\;\left(1-\pi_{2}\right)\right]\left[1+\left(m-1\right)\;\rho \right]}{{\left(\pi_{1}-\pi_{2}\right)}^{2}}$$

with a type I error of 5% (α = 0.05) and 90% power (β = 0.10). There will be 290 subjects included in total, allowing for a dropout rate of 10%.

### Randomization and blinding

Each participating CHC will be assigned to either experimental group or control group by restricted randomization generated by an independent statistician, who works in the evidence-based center of FJTCM, via SAS software (Version 8.2, SAS Institute, Cary, North Carolina, United States). The random allocation sequence is blinded to the screeners who make the baseline test for the patients, and protected by a specified project manager who is not involved in the recruitment program of this study. Both allocation and baseline measurements will be concealed to outcome assessors.

### Baseline tests

Baseline descriptive data will be obtained using a questionnaire consisting of the demographic data of subjects, such as gender, age, height, weight, nationality, education level, employment status, and information about stroke including the type, duration and location. All candidates will be tested on the severity of aphasia using the Boston diagnostic aphasia examination- Chinese version (BDAE-C). Broca’s aphasia is ascertained by CRRCAE. The functional condition of the brain is examined by using blood oxygen level dependent-functional magnetic resonance imaging (BOLD-*f*MRI) examination. Activities of daily life (ADL) will be reported with the Modified Barthel Index (MBI) and quality of life will be collected with the 36-Item Short Form Health Survey (SF-36).

### Intervention

Participants in the experimental group will receive integrated acupuncture therapy with SLT and initial therapies, meanwhile the control group will only receive SLT and initial therapy. The initial therapy consists of health education and conventional medical therapy. The treatments will take place five times per week for 4 weeks in total. All of the employees participated in this trial will be standardized in their training, including in the study protocol, methods of treatments, and assessments.

### Health education

For the purposes of improving patients’ knowledge of stroke and teaching them about the harmfulness of stroke and the necessity of treatment, patients admitted to hospital will be provided with access to educational brochures on stroke, which are based on the blueprint of ‘Freedom from the Misunderstanding of Stroke Rehabilitation’ (the first prize among popular science books from the Chinese Association of TCM) [24]. Information about stroke in these brochures will include the concept, epidemiology, etiology and pathogenesis, risk factors, common symptoms, diagnosis, common complications, treatments, and precautions that should be taken. At the same time, rehabilitation physicians will introduce themselves and explain about the contents of the educational brochures to all participants in person.

### Conventional medical therapy

All participants will receive conventional medical therapies according to the Guidelines for the Prevention and Treatment of Cerebrovascular Disease in China (2007) published by the Chinese Medical Association [25]. Specific treatments involve the interventions and management of the causes of disease, risk factors, and a range of comorbid conditions such as heart disease, diabetes, and hypertension.

### Speech and language therapy

During a retained time of the scalp acupuncture therapy, the experimental group patients will receive SLT which is based on the ‘Technical specification of Common rehabilitation therapy’ (2012) published by Chinese Association of Rehabilitation Medicine [26]. The therapists will prescribe appropriate training programs, including different aspects of listening, speaking, reading, writing, or calculating and so on, on the evidence of each patient’s assessment result. Furthermore, the training’s degree of difficulty is based on every patient’s progress. The period of treatment will be continuous for 4 weeks at 5 days a week and 30 minutes per day.

### Scalp acupuncture therapy

The lower 2/5 of the anterior oblique line of vertex-temporal MS 6 (*Ding nie qian xie xian*) and anterior temporal line MS 10 (*Nie qian xian*) will be the needling sites. The anterior oblique line of vertex-temporal, which links EX-RN 1 (*Qian Si shen cong*) to GB 6 (*Xuan li*), is on the temporal side of the head and obliquely passes through the bladder and gallbladder meridians. The anterior temporal line is a part of the gallbladder meridian on the temporal side of the head, linking GB 4 (*Hanyan*) to GB 6 (*Xuanli*).

For correct manipulation, either a sitting or a dorsal position is appropriate. The area should be routinely sterilized before insertion of the needle. Number 30 Hwato needles of 0.30 mm in diameter and 40 mm in length (Suzhou Medical Appliance Factory, China) will be obliquely inserted (10 to 20° into the scalp) into the gales aponeurotica rapidly, then parallelly pierced 25 to 35 mm deep, along the lines described above, for point prescription. The acupuncture will be maintained for 30 minutes with rapid twirling performed for 2 to 3 minutes once 10 minutes, at a frequency of 180 to 300 twirls per minute. Each time after withdrawing the needle, a dry sterilized cotton ball should be pressed the puncture holes as quickly as possible to prevent bleeding. The therapy will be given once a day for 5 days a week lasting for 4 weeks continuously.

### Follow-up

There is a period of 12-week follow-up after the 4-week treatment. During this time trained personnel will call the participants and take a record every week, asking them something about their rehabilitation situation and whether they have received other treatments. Home visits will be at least once a month to assess their functional status including speech function, activities of daily living, and quality of life, using CRRCAE, BDAE-C,MBI, and SF-36 after 6 and 12 weeks follow-up.

### Outcome measures

Except for baseline (-2 to -1 weeks), all of the primary and secondary outcomes assessment will be also conducted at end of 2-week treatment (3 weeks), end of 4-week treatment (5 weeks), end of 6-week follow-up (11 weeks), and end of follow-up period (17 weeks) with a BOLD-*f*MRI examination only at end of the 4-week treatment (5 weeks). A summary of all the measures in the trial is shown in Table 1.

**Table 1:** Trial processes chart

**Items**		**Before enrolment (weeks) -2 to (-1)**	**Intervention period (weeks) 1 to 2**	**End of 2-week treatment (weeks) 3**	**Intervention period (weeks) 3 to 4**	**End of 4-week treatment (weeks) 5**	**Follow-up (weeks) 5 to 10**	**End of 6-week follow-up (weeks) 11**	**Follow-up (weeks) 11 to16**	**End of 12-week follow-up (weeks) 17**
Recruitment		**×**								
Enrolment		**×**								
Inclusion criteria		**×**								
Exclusion criteria		**×**								
Informed consent		**×**								
Basic characteristic variables		**×**								
Randomization and allocation concealment		**×**								
Primary outcomes	Speech function	**×**		**×**		**×**		**×**		**×**
	Severity of aphasia	**×**		**×**		**×**		**×**		**×**
Secondary outcomes	Activities of daily life	**×**		**×**		**×**		**×**		**×**
	Quality of life	**×**		**×**		**×**		**×**		**×**
	Functional condition of the brain	**×**				**×**				
Adverse events recorded			**×**		**×**		**×**		**×**	
Self-reported drug therapy			**×**		**×**		**×**		**×**	
Cost sheet of rehabilitation therapy			**×**		**×**		**×**		**×**	

### Primary outcomes

The primary outcome measures in this study are the differences in the scores on both the CRRCAE and the aphasia grading standard of severity in BDAE-C after intervention and follow-up between the two groups and within groups.

The CRRCAE [27,28] was made by Chinese Rehabilitation Research Center with the help of Japanese experts according to Chinese language and cultural habits by referring to the Japanese Standard language Test of Aphasia (SLTA). It consists of two parts. One part includes twelve questions about demography date and is used to test the patients’ general condition of speech. The other part contains nine subtests (auditory comprehension, repetition, speaking, reading aloud, reading comprehension, copying, description, dictation, and calculation). There are 30 quizzes in total, with three or four quizzes in each subtest. This scale has been used widely in hospitals and rehabilitation centers in China with a good reliability and validity. The interclass correlation coefficients (ICC) of test-retest reliability exceeded 0.9, the Cronbach’s α of internal consistency was 0.941, and the relationship with the Western Aphasia Battery (WAB) was 0.948 (on aphasia quotients). Furthermore, we can diagnose whether a person has aphasia and, if so, the degree of severity from the result of CRRCAE which can guide the strategies of therapy and can also measure the outcomes of treatment.

BDAE [29] is a widely used aphasia examination in the world. As the BDAE original edition is not suitable for Chinese conditions, it was revised to BDAE-C [30,31] based on the Chinese culture and language background, and is now established as the standard for Chinese patients. There are six grades in the aphasia grading standard of severity in BDAE-C from the worst to the best in the order of 0 to 5.

### Secondary outcomes

The differences on the MBI, SF-36, and the results of BOLD-*f*MRI examination after intervention are considered as the secondary outcome measures.

The MBI [32] is a widely used tool, measuring the extent to which somebody can function independently and have mobility in their activities of daily living (ADL), and also indicating the need for assistance in care. It contains 10 items and is scored on a scale from 0 to 100 with a higher score indicating more independence [33].

The SF-36 [34] is a short-form health survey with 36 questions. It yields an 8-scale profile of functional health and wellbeing scores, as well as psychometrically-based physical and mental health summary measures and a preference-based health utility index. It is a generic measure, regardless of age, disease, or treatment group. The SF-36 has proven useful in differentiating the health benefits produced by a wide range of different treatments.

The BOLD-*f*MRI is the primary form of functional magnetic resonance imaging, and it is a type of specialized brain scan used to map neural activity in the brain of humans or other animals by imaging the change in blood flow (hemodynamic response) related to energy use by brain cells [35]. The procedure is similar to MRI but uses the change in magnetization between oxygen-rich and oxygen-poor blood as its basic measure. The resulting brain activation can be presented graphically by color-coding the strength of activation across the brain or the specific region studied. The technique can also localize activity to within millimeters.

### Safety assessments

All of the adverse events (AEs) that happen during the treatment and follow-up period will be recorded in detail by researchers and therapists at each CHC. Serious adverse events will be reported to the Human Research Ethics Committee (HREC) of FJTCM immediately, who will make a judgment on whether the participant can continue in the trial depending on the patient’s condition. Any adverse event will be analyzed regardless of the causality related to intervention and will receive corresponding treatment measures.

### Economic evaluation

The indicator of economic evaluation will employ a cost-effectiveness ratio, meaning the required cost of every point increased on the CRRCAE. The cost of interventions consists of direct costs and indirect costs. Direct costs will include any expense involving treatments throughout the rehabilitation period. The number of workdays lost by participants and care-givers owing to illness will be counted in indirect costs. Finally, there will be a comparison on the differences of cost-effectiveness ratio between CHCs and high-grade hospitals.

### Statistical methods

Statistical analysis in this study will be performed by a statistician who is not involved in this trial and works in the Center of Evidence-based Medicine in FJTCM. Analysis of all data will be conducted with SAS software (version 8.2, SAS Institute, Cary, North Carolina, United States), and two-sided *P* < 0.05 was considered statistically significant.

Descriptive statistics will be expressed in different forms according to the types of the data in each group. That is to say, continuous variables will use mean and standard deviation for normal distribution, with median and interquartile range for non-normal distribution, and categorical variables will use proportions with their standard error. Comparison of baseline characteristics, the primary or secondary outcomes between groups will use the *t*-test or Mann-Whitney test for continuous variables and Pearson chi-squared or Fisher’s exact test for categorical variables. Changes in effect sizes of primary or secondary outcomes from baseline to each time point for two groups will be expressed using the paired *t*-test or Wilcoxon signed-rank test.

The primary outcomes will be analyzed using full analysis set (FAS) and per protocol set (PPS), with the secondary outcomes using FAS. Analysis of the primary and secondary outcomes should be on the basis of the intention-to-treat (ITT) population. The safety set (SS) will be used to assess adverse events.

## Discussion

Nearly 20 to 25% of all stroke patients suffer from aphasia [36], and approximately half of the initially affected patients still suffer from aphasia one year after stroke [10,12]. Acupuncture, which proved to have a positive therapeutic effect on aphasia in acute stage stroke patients [37], is considered as a good choice for chronic stroke patients with aphasia searching for complementary and alternative medicine as longterm treatment at a low cost.

We adopt a randomized, parallel-controlled, single-blind design in this trial to guarantee the quality level of the study. In consideration of the characteristics of stroke-induced aphasia, we will investigate the value-in-apply of combined SLT to scalp acupuncture treatment from the view of safety and efficacy, by the way of comparing the cost-effectiveness ratio of aphasia rehabilitation between CHCs and high-grade hospitals. We will also judge the longterm stability of improvements by a 12-week follow-up after intervention and dynamically observe the changes of curative effects by setting five assessment points (baseline, 2 and 4 weeks after intervention,6 and 12 weeks after follow-up).

A number of studies have come to an agreement that aphasia treatment is effective in enhancing recovery [38-41]. However, there is a controversy about the role of the course of disease on outcomes. Some trials hold the view that further improvement of language abilities is very limited beyond 6 months after the onset of stroke [42] and therapy provided immediately after stroke results in more beneficial effects than deferred treatment [43].Others think that language abilities can be significantly improved in the chronic stage after a stroke when training is sufficiently intensive [44-47]. In the present trial, if possible, we intend to make a stratified analysis according to the course of the disease to discover the best time to start treatment. In addition, cortical plasticity is recognized as the main mechanism of functional recovery basing on neuroimaging findings [48-50]. In this study, we will use *f*MRI to observe the changes of activation of brain hemisphere structures attributed to the treatment.

A limitation of this study is that this protocol is not double-blind and has no placebo control. However, the intervention group will be blinded to the evidence-based medicine center to decrease possible bias. The study also cannot eliminate the confounding effects from the spontaneous recovery in the early stage of stroke.

If successful, this project will offer a low-cost appropriate technology for the CHCs and could be implemented on a large scale, both in China and worldwide. Scalp acupuncture therapy, which can be performed in a CHC instead of in a high-grade hospital, will relieve the stress of hospitalization and reduce the financial burden for the patient and healthcare system.

## Trial status

This trial started in November 2013 and the last patient will be included on August 1, 2015.

## Abbreviations

ADL: Activities of daily life; AE: Adverse event; BDAE-C: Boston diagnostic aphasia examination–Chinese version; BOLD-*f*MRI: Blood-oxygen-level dependent functional magnetic resonance imaging; CHC: Community health center; CRRCAE: Chinese Rehabilitation Research Center Standard Aphasia Examination; CT: Computed tomography; FAS: Full analysis set; FJTCM: Fujian University of Traditional Chinese Medicine; HREC: Human Research Ethics Committee; ICC: Interclass correlation coefficients; ITT: Intention-to-treat; LOS: Length of stay; LOS-Eff: LOS efficiency; MBI: Modified Barthel Index; MRI: Magnetic resonance imaging; PPS: Per protocol set; SF-36: 36-item short form health survey; SLT: Speech and language therapy; SLTA: Japanese Standard language Test of Aphasia; SS: Safety set; WAB: Western Aphasia Battery.

## Competing interests

The authors declared that they have no competing interests.

## Authors’ contributions

CLD, TJ, YSL and ZGH conceived of the study, designed the study protocol, and drafted the manuscript. FYH, WZK wrote the manuscript. TJ revised study protocols and wrote several sections of the manuscript. TJ is in charge of coordination and direct implementation. RT, SYS, WJS, LLL and LW helped to develop the study measures and analyses. All authors contributed to drafting the manuscript and have read and approved the final manuscript.

## References

[B1] Tilling K Sterne JA Rudd AG Glass TA Wityk RJ Wolfe CD A new method for predicting recovery after stroke Stroke 2001 32 2867 2873 11739989 10.1161/hs1201.099413

[B2] Paolucci S Antonucci G Pratesi L Traballesi M Lubich S Grasso MG Functional outcome in stroke inpatient rehabilitation: predicting no, low and high response patients Cerebrovasc Dis 1998 8 228 234 9684063 10.1159/000015856

[B3] Gialanella B Prometti P Rehabilitation length of stay in patients suffering from aphasia after stroke Top Stroke Rehabil 2009 16 437 444 20139046 10.1310/tsr1606-437

[B4] Bjorkdahl A Sunnerhagen KS Process skill rather than motor skill seems to be a predictor of costs for rehabilitation after a stroke in working age; a longitudinal study with a 1 year follow up post discharge BMC Health Serv Res 2007 7 209 18154643 10.1186/1472-6963-7-209 PMC2265694

[B5] Hofgren C Bjorkdahl A Esbjornsson E Sunnerhagen KS Recovery after stroke: cognition, ADL function and return to work Acta NeurolScand 2007 115 73 80 10.1111/j.1600-0404.2006.00768.x 17212608

[B6] Black-Schaffer RM Osberg JS Return to work after stroke: development of a predictive model Arch Phys Med Rehabil 1990 71 285 290 2327878

[B7] Hilari K Northcott S Roy P Marshall J Wiggins RD Chataway J Ames D Psychological distress after stroke and aphasia: the first six months Clin Rehabil 2010 24 181 190 20103578 10.1177/0269215509346090

[B8] Wade DT Hewer RL David RM Enderby PM Aphasia after stroke: natural history and associated deficits J Neurol Neurosurg Psychiatry 1986 49 11 16 2420939 10.1136/jnnp.49.1.11 PMC1028640

[B9] Kwa VI Limburg M de Haan RJ The role of cognitive impairment in the quality of life after ischaemic stroke J Neurol 1996 243 599 604 8865027 10.1007/BF00900948

[B10] Laska AC Hellblom A Murray V Kahan T Von Arbin M Aphasia in acute stroke and relation to outcome J Intern Med 2001 249 413 422 11350565 10.1046/j.1365-2796.2001.00812.x

[B11] Wendt O Koul R Hassink JM Time post-onset does not affect response to treatment in patients with chronic aphasia ≥ 1 year after stroke 1 Evid Based Commun Assess Interv 2008 2 199 202

[B12] Pedersen PM Vinter K Olsen TS Aphasia after stroke: type, severity and prognosis. The Copenhagen aphasia study Cerebrovasc Dis 2004 17 35 43 14530636 10.1159/000073896

[B13] Brady MC Kelly H Godwin J Enderby P Speech and language therapy for aphasia following stroke Cochrane Database Syst Rev 2012 CD000425 22592672 10.1002/14651858.CD000425.pub3

[B14] Sun Y Xue SA Zuo Z Acupuncture therapy on apoplectic aphasia rehabilitation J Tradit Chin Med 2012 32 314 321 23297549 10.1016/s0254-6272(13)60031-x

[B15] Nouwens F Dippel DW de Jong-Hagelstein M Visch-Brink EG Koudstaal PJ de Lau LM Rotterdam Aphasia Therapy Study (RATS)-3: "The efficacy of intensive cognitive-linguistic therapy in the acute stage of aphasia"; design of a randomised controlled trial Trials 2013 14 24 23343197 10.1186/1745-6215-14-24 PMC3560268

[B16] Elsner B Kugler J Pohl M Mehrholz J Transcranial direct current stimulation (tDCS) for improving aphasia in patients after stroke Cochrane Database Syst Rev 2013 6 CD009760 23799617 10.1002/14651858.CD009760.pub2

[B17] Kaptchuk TJ Acupuncture: theory, efficacy, and practice Ann Intern Med 2002 136 374 383 11874310 10.7326/0003-4819-136-5-200203050-00010

[B18] Pang Y Wu LB Liu DH Acupuncture therapy for apoplectic aphasia: a systematic review Zhongguo Zhen Jiu 2010 30 612 616 20862949

[B19] Liu L Wang D Wong KS Wang Y Stroke and stroke care in China: huge burden, significant workload, and a national priority Stroke 2011 42 3651 3654 22052510 10.1161/STROKEAHA.111.635755

[B20] Klebic J Salihovic N Softic R Salihovic D Aphasia disorders outcome after stroke Med Arh 2011 65 283 286 22073852 10.5455/medarh.2011.65.283-286

[B21] Lui MH Ross FM Thompson DR Supporting family caregivers in stroke care: a review of the evidence for problem solving Stroke 2005 36 2514 2522 16210553 10.1161/01.STR.0000185743.41231.85

[B22] The Fourth Academic Seminar of the Chinese Society for Neuroscience Major diagnostic points of cerebrovascular disease Chin J Neurol 1996 29 379 380

[B23] Zhang HM Clinical treatment of apoplectic aphemia with multi-needle puncture of scalp-points in combination with visual-listening-speech training Zhen Ci Yan Jiu 2007 32 190 194 17691578

[B24] Chen LD Freedom from the Misunderstanding of Stroke Rehabilitation 2008 Beijing: People’s Medical Publishing House

[B25] Rao ML Guidelines for the Prevention and Treatment of Cerebrovascular Disease in China 2007 Beijing: People’s Medical Publishing House

[B26] Chinese Association of Rehabilitation Medicine Technical Specification of Common Rehabilitation Therapy 2012 Beijing: People’s Medical Publishing House

[B27] Zhang QS Ji SR Li SL He Y Jia GH Qin JT Wei DJ Tian H Reliability and validity of Chinese rehabilitation research center standard aphasia examination Chin J Rehabil Theory Practice 2005 11 703 705

[B28] Li SL Xiao L Tian H Wei DJ Qin JT Feng DX Jia GH Chen W He Y Zhang QS Li Z Zhu LJ Qiu WH Wu ZH Wang QB Zhu XF Lei B Wang J Zhu YP Wang CP Lu M Introduction to Chinese standard aphasia examination Chin J Rehabil Theory Practice 2000 6 162 164

[B29] Goodlass H Kaplan E The assessment of aphasia and related disorders Hecaen and Albert 1979 1982 Philadelphia: Lea & Febiger

[B30] Wang J Zhang P Research on Boston diagnostic aphasia examination-Chinese version and its norm Zhong Guo Kang Fu 1996 11 49 51

[B31] Wang J Lv YL Reliability of Boston diagnostic aphasia examination-Chinese version Zhong Guo Kang 1998 13 121 122

[B32] Leung SO Chan CC Shah S Development of a Chinese version of the Modified Barthel Index–validity and reliability ClinRehabil 2007 21 912 922 10.1177/0269215507077286 17981850

[B33] Sibbritt D van der Riet P Dedkhard S Srithong K Rehabilitation of stroke patients using traditional Thai massage, herbal treatments and physical therapies Zhong Xi Yi Jie He Xue Bao 2012 10 743 750 22805080 10.3736/jcim20120704

[B34] Ware JJ Sherbourne CD The MOS 36-item short-form health survey (SF-36). I. Conceptual framework and item selection Med Care 1992 30 473 483 1593914

[B35] Huettel SA Song AW McCarthy G Functional Magnetic Resonance Imaging 2009 Massachusetts: Sinauer Associates Sunderland

[B36] Vaatjes I van Dis I Visseren FLJ Bots ML Vaatjes I, van Dis I, Visseren FLJ, Bots ML Cardiovascular Diseases in the Netherlands in Women and men Cardiovascular Diseases in The Netherlands in 2011. Facts on Lifestyle and Risk Factors 2011 Den Haag: Dutch Heart Foundation 7 22

[B37] Li JA Clinical observation on acupuncture for treatment of aphasia due to ischemic stroke at the early stage Zhongguo Zhen Jiu 2005 25 760 762 16335197

[B38] Cao Y Vikingstad EM George KP Johnson AF Welch KM Cortical language activation in stroke patients recovering from aphasia with functional MRI Stroke 1999 30 2331 2340 10548667 10.1161/01.str.30.11.2331

[B39] Heiss WD Kessler J Thiel A Ghaemi M Karbe H Differential capacity of left and right hemispheric areas for compensation of poststroke aphasia Ann Neurol 1999 45 430 438 10211466 10.1002/1531-8249(199904)45:4<430::aid-ana3>3.0.co;2-p

[B40] Karbe H Thiel A Weber-Luxenburger G Herholz K Kessler J Heiss WD Brain plasticity in poststroke aphasia: what is the contribution of the right hemisphere? Brain Lang 1998 64 215 230 9710490 10.1006/brln.1998.1961

[B41] Warburton E Price CJ Swinburn K Wise RJ Mechanisms of recovery from aphasia: evidence from positron emission tomography studies J NeurolNeurosurg Psychiatry 1999 66 155 161 10.1136/jnnp.66.2.155 PMC1736204 10071093

[B42] Carlomagno S Pandolfi M Labruna L Colombo A Razzano C Recovery from moderate aphasia in the first year poststroke: effect of type of therapy Arch Phys Med Rehabil 2001 82 1073 1080 11494187 10.1053/apmr.2001.25155

[B43] Teasell R Bitensky J Salter K Bayona NA The role of timing and intensity of rehabilitation therapies Top Stroke Rehabil 2005 12 46 57 16110427 10.1310/ETDP-6DR4-D617-VMVF

[B44] Barthel G Meinzer M Djundja D Rockstroh B Intensive language therapy in chronic phasia: which aspects contribute most? Aphasiology 2008 22 408 421

[B45] Meinzer M Djundja D Barthel G Elbert T Rockstroh B Long-term stability of improved language functions in chronic aphasia after constraint-induced aphasia therapy Stroke 2005 36 1462 1466 15947279 10.1161/01.STR.0000169941.29831.2a

[B46] Richter M Miltner WH Straube T Association between therapy outcome and right-hemispheric activation in chronic aphasia Brain 2008 131 1391 1401 18349055 10.1093/brain/awn043

[B47] Pulvermuller F Neininger B Elbert T Mohr B Rockstroh B Koebbel P Taub E Constraint-induced therapy of chronic aphasia after stroke Stroke 2001 32 1621 1626 11441210 10.1161/01.str.32.7.1621

[B48] Chau AC Fai CR Jiang X Au-Yeung PK Li LS An fMRI study showing the effect of acupuncture in chronic stage stroke patients with aphasia J Acupunct Meridian Stud 2010 3 53 57 20633517 10.1016/S2005-2901(10)60009-X

[B49] Li G Yang ES An fMRI study of acupuncture-induced brain activation of aphasia stroke patients Complement Ther Med 2011 19 Suppl 1 S49 S59 21195295 10.1016/j.ctim.2010.11.004

[B50] Heath S McMahon KL Nickels L Angwin A Macdonald AD van Hees S Johnson K McKinnon E Copland DA Neural mechanisms underlying the facilitation of naming in aphasia using a semantic task: an fMRI study BMC Neurosci 2012 13 98 22882806 10.1186/1471-2202-13-98 PMC3477078

